# Changes in White Matter Integrity before Conversion from Mild Cognitive Impairment to Alzheimer’s Disease

**DOI:** 10.1371/journal.pone.0106062

**Published:** 2014-08-25

**Authors:** Michaela Defrancesco, Karl Egger, Josef Marksteiner, Regina Esterhammer, Hartmann Hinterhuber, Eberhard A. Deisenhammer, Michael Schocke

**Affiliations:** 1 Department of General Psychiatry, Innsbruck Medical University, Innsbruck, Austria; 2 Department of Radiology I, Innsbruck Medical University, Innsbruck, Austria; 3 Department of Psychiatry, LKH Hall, Hall, Austria; 4 Department of Neuroradiology, University Clinic Freiburg, Freiburg, Germany; University of Barcelona, Spain

## Abstract

**Background:**

Mild cognitive impairment (MCI) may represent an early stage of dementia conferring a particularly high annual risk of 15–20% of conversion to Alzheimer’s disease (AD). Recent findings suggest that not only gray matter (GM) loss but also a decline in white matter (WM) integrity may be associated with imminent conversion from MCI to AD.

**Objective:**

In this study we used Voxel-based morphometry (VBM) to examine if gray matter loss and/or an increase of the apparent diffusion coefficient (ADC) reflecting mean diffusivity (MD) are an early marker of conversion from MCI to AD in a high risk population.

**Method:**

Retrospective neuropsychological and clinical data were collected for fifty-five subjects (MCI converters n = 13, MCI non-converters n = 14, healthy controls n = 28) at baseline and one follow-up visit. All participants underwent diffusion weighted imaging (DWI) and T1-weighted structural magnetic resonance imaging scans at baseline to analyse changes in GM density and WM integrity using VBM.

**Results:**

At baseline MCI converters showed impaired performance in verbal memory and naming compared to MCI non-converters. Further, MCI converters showed decreased WM integrity in the frontal, parietal, occipital, as well as the temporal lobe prior to conversion to AD. Multiple regression analysis showed a positive correlation of gray matter atrophy with specific neuropsychological test results.

**Conclusion:**

Our results suggest that additionally to morphological changes of GM a reduced integrity of WM indicates an imminent progression from MCI stage to AD. Therefore, we suggest that DWI is useful in the early diagnosis of AD.

## Introduction

Alzheimer’s disease (AD) is a progressive neurodegenerative disorder associated with loss of memory and other cognitive functions. The concept of “mild cognitive impairment” has been introduced to individuals not yet fulfilling the criteria of AD but whose memory and current cognitive profile is abnormal in comparison with their contemporaries [1]–[3]. The annual conversion rate of patients with MCI to AD is approximately 15%–20% per year, whereas conversion rates of 1–2% per year are reported in healthy elderly individuals [5]. Recent research provide increasing evidence that pathophysiological changes associated with AD begin 10 to 25 years before clinical dementia onset and are possibly already detectable in MCI stage [6]–[10]. Therefore, the identification of clinical, as well as neurobiological markers, in the early diagnosis of AD may improve diagnostic accuracy and enables early effective treatment. Consequently, there is an urgent need to identify associated biomarkers that can help to diagnose AD in the preclinical and early clinical stages [11]. A recent review by Gold et al. 2012 reported that WM integrity in a subset of tracts declines already in pre-clinical stages of AD [12]. Prior studies comparing converting and non-converting MCI patients revealed increased atrophy of gray matter and a higher load of white matter lesions in pre-clinical AD [13], [14]. However, results of prior studies using different neuroimaging methods in preclinical dementia are not uniform and often fail to provide longitudinal data. Thus, there is an emerging consensus that ongoing AD research should focus on the detection and evaluation of pre-symptomatic imaging biomarkers [15], [16].

The essentially pathological signs of AD are extracellular amyloid-β protein deposits and intracellular neurofibrillary tangles on a molecular level, which occur together with structural changes such as atrophy of cortical and sub-cortical structures [17]–[19]. Previous positron emission tomography (PET) studies have shown that the probability for conversion also depends on the load of amyloid-β protein deposits [20], whereby 20–30% of the normal controls without any cognitive deficits also exhibited β -amyloid protein deposits [21], [11].

Although the main pathology of AD involves changes in cortical GM structures such as the hippocampus, there is evidence that sub-cortical structures like the nucleus caudatus are involved in the pathophysiology of AD, too [22]. Group differences in atrophy patterns of patients with AD and MCI have been investigated by numerous previous VBM studies; these included in particular GM density in normal aging [23], mild Alzheimer’s disease [24], and MCI- patients [25]. Existing data provide evidence that VBM can distinguish between healthy aging and AD, as well as predict conversion from MCI to AD with high sensitivity [26], [27]. The results of a recent VBM study indicate that MCI patients exhibit similar atrophy patterns - predominately of the hippocampal region - as AD patients one year prior to conversion [25]. Further, VBM-measured volume loss in the entorhinal cortex allowed a discrimination of amnestic MCI and/or early Alzheimer’s disease from healthy age- and gender-matched controls with an discrimination accuracy up to 87.8% [28].

Diffusion-weighted imaging (DWI) measures disruption of cellular integrity of nerve fibres caused by neuronal loss in AD pathology. These disruptions cause a higher degree of water molecule motion and, consequently, an increase of the apparent diffusion coefficient (ADC) [29].

A study of Cherubini et al. [30] using DWI reported increased mean diffusivity in the hippocampus, the amygdale, and the caudate nucleus in MCI and AD patients compared to healthy controls. Other prior studies using DWI compared the ADC of MCI patients and healthy controls and found higher ADC values in the hippocampus, corpus callosum and temporal lobe in MCI patients [30], [31]. Kantarci et al. [32] reported that higher ADC values in the hippocampus in MCI patients are associated with a higher risk of conversion to AD.

VBM is a reliable method for comparing typical atrophy-patterns of GM and WM of different groups of patients. Further, VBM can be used for the evaluation of T1-weighted 3D volume sets or ADC maps without requiring a-priori-hypothesis. To date, studies comparing diffusion-weighted images in MCI converters and MCI non-converters using the methodology of VBM are rare and do not provide longitudinal follow-up data. The purpose of our study was to find typical patterns of GM atrophy, as well as pathological changes in WM integrity in MCI patients prior to conversion from MCI to AD. In terms of developing therapeutic methods that modify or even stop the course of AD, early identification of subjects who will convert to AD is of great scientific interest. Further, a purpose of this study was to correlate neuroimaging findings with neuropsychological results from a detailed neuropsychological assessment such as the CERAD battery [33] in MCI patients and healthy controls.

## Materials and Methods

### Study design and ethics

This study was approved by the Ethics Committee of Innsbruck Medical University, Austria. Written informed consent was given by all participants for their clinical records to be used in this study.

This was a retrospective, observational study to assess the impact of neuropsychological and structural changes of GM and WM integrity on the imminent conversion from MCI to AD. Data of fifty-five patients were retrospectively analysed from files of the outpatient memory clinic of the University Clinic of Innsbruck between 2005 and 2011 at two time points. MCI converters, MCI non-converters and healthy controls were retrospectively selected out of archived medical histories at out memory clinic based on age, gender, education and comparable comorbidities. All patients completed a neuropsychological assessment and a clinical interview at baseline and follow-up visit within their routine assessment at our outpatient clinic. Inclusion criteria for this study were: German speaking, age≥60 years old, stable and controlled medical conditions such as hypertension, diabetes mellitus and hyperlipidemia. Subjects were excluded if they had neurological diseases including stroke, severe small vessel disease (subcortical arteriosclerotic encephalopathy) defined as a Fazekas score for WM lesions >1 [34], Parkinson’s disease, normal pressure hydrocephalus or a serious systematic major disease (i.e. cardiac disease, current infection, significant hepatic, renal, pulmonary, metabolic or endocrine disturbances), were currently diagnosed with a psychiatric disorder (including dementia, affective disorders and schizophrenia), or had metal in the body that precluded magnetic resonance imaging (MRI). MCI (amnestic MCI or amnestic MCI multi-domain Type) was diagnosed according to the criteria of Petersen et al. [3]. Further all MCI patients had to report subjective memory complaints over the last 6 month, had a Clinical Dementia Rating Scale (CDR) score [35] of 0.5, and impaired memory function (verbal of figural) in neuropsychological assessment >1.5 standard deviation (SD) related to age and education. At follow-up, AD was clinically diagnosed according to the NINCDS-ADRDA Alzheimer’s Criteria proposed in 1984 by McKhann (Probable Alzheimer’s disease) [4] using the same clinical interview and neuropsychological assessments. Progression from MCI to AD was defined as: percent range of 5 or lower corrected for age and education in neuropsychological assessment of at least one memory function (verbal or figural memory), presents of subjective memory complaints over the last 6 month, deficits in activities of daily living assessed by a clinical interview, and a CDR score of >0.5. No patient was diagnosed as having AD or received an antidementive medication prior to follow-up visit. Neuropsychological testing.

All subjects also underwent a detailed neuropsychological assessment at baseline and follow-up. They performed the german Version of the Mini-Mental State Examination (MMSE) [36] and were tested on verbal memory (word list learning, free recall) and recognition subtests of the CERAD battery [33], figural memory (free recall, CERAD), object naming (Boston Naming Test – short version, CERAD), categorical verbal fluency (animals/min, s-words/min CERAD), planning (CLOX Test part 1), constructive abilities (copy geometrical shapes, CERAD), and divided attention and cognitive flexibility (Trail Making Test-A) [37].

All participants completed the Geriatric Depression Scale (GDS) [38].

### MRI data acquisition

MRI scans were conducted within 2 months of clinical and neuropsychological examination. All MRI data were obtained using a 1.5 Tesla whole-body MR scanner (Magnetom Symphony, Siemens Erlangen, Germany) and a circular-polarized head coil. The MR protocol comprised a sagittal magnetization prepared rapid acquisition gradient echo (MP-RAGE) sequence with a repetition time (TR) of 1500 ms, an echo time (TE) of 4.38 ms, an inversion time (TI) of 800 ms, a field of view (FoV) of 230×230 mm, a matrix of 256×256, a slice thickness of 1.2 mm, and a flip angle of 15°. The diffusion-weighted sequence had diffusion-sensitizing gradients in six directions with b-factors of 0 and 1000 s2/mm, a TR of 6000 ms, a TE of 96 ms, a FoV of 230×230 mm, a matrix of 128×128 (Fourier-interpolated to 256×256 during acquisition), a flip angle of 90°, a slice thickness of 3 mm, and a gap of 0.75 mm. The brain was covered by 35 slices. In addition, we performed a transversal double-echo turbo spin echo sequence with a TR of 4050 ms, TEs of 16 and 104 ms, a slice thickness of 4 mm, a gap of 1 mm, a flip angle of 150°, a FoV of 220×187 mm, and a matrix of 256×173 as well as a coronal turbo inversion recovery sequence with a TR of 9000 ms, a TE of 114 ms, a TI of 2200 ms (dark-fluid preparation), an echo train length of 21, a FoV of 230×180 mm, a matrix of 256×200, a slice thickness of 5 mm, and a gap of 1 mm.

Maps of the apparent diffusion coefficient (ADC) were calculated by using the Neuro3D card of the MR scanner and averaging over six directions. Consequently, the resulting ADC maps reflected the mean diffusivity (MD) [39]. Artifacts and eddy current distortions were visually and qualitatively estimated as previous described [40]. Data sets with substantial artifacts or distortions were excluded from further analyses. All data sets were stored in the PACS system using the DICOM standards.

A radiologist viewed all MR-images for structural abnormalities inconsistent with diagnosis, as well as artefacts which could compromise downstream operations using VBM.

### Voxel-based Morphometry

T1 and diffusion weighted images were processed using SPM8 software (Wellcome Department of Imaging Neuroscience Group, Institute of Neurology, London) running with MATLAB 7.11 R2010b (Mathworks Inc., Natick, MA, USA). VBM analysis were conducted using the VBM toolbox (Christian Gaser, http://dbm.neuro.uni-jena.de/vbm.html) with default parameters. Further, the Diffeomorphic Anatomical Registration Through Exponentiated Lie Algebra (DARTEL) toolbox was used to generate a series of customized templates and flow fields of GM and WM images by performing iterative registration [41]. First, raw data in DICOM (.dcm) format were imported into SPM. The maps of GM and WM were then spatially normalized to a group specific template (of all subjects) in MNI space using the DARTEL toolbox. All images were normalized to the standard MNI template and segmented into GM, WM, and cerebrospinal fluid. The resulting maps were smoothed with an 8 mm full-width half maximum (FWHM) Gaussian Kernel to increase the signal to noise ratio. The smoothed GM images were entered into a statistical model (t-test, multiple regression), estimated, and a contrast was defined.

Because ADC values do not compartmentalize in GM and WM, analysis on the whole brain were performed without segmentation. First, ADC maps reflecting MD were calculated by the software of the MRI scanner (1.5 Tesla whole-body MR scanner - Magnetom Symphony, Siemens Erlangen, Germany) and exported in dicom. format. Secondly, dicom’s were imported in SPM using the “import image” function. Next, all diffusion weighted images were in a first step co-registered with the corresponding high resolution T1 images. Then, ADC maps were spatially normalized to MNI space by applying the warps of the T1 image. Finally, these normalized ADC maps were smoothed and entered into a statistical model.

### Statistical analysis

Statistical comparison of demographic data between the three groups (healthy controls, MCI-converters, MCI non-converters) were performed at baseline using analysis of variance (one-way ANOVA). To test for changes in neuropsychological measures in the three diagnostic groups between baseline and follow-up measuring the Wilcoxon Matched-Pairs Test was used.

To investigate differences in neuropsychological measures between the three diagnostic groups ab baseline and follow-up visit repeated-measures analysis of variance (ANOVA) was used, with the diagnostic group (3 levels) and time (2 levels) as within-subject factors. The Huynh-Feldt method was used to account for non-sphericity. To adjust for possible confounding variables age, education and sex were added as covariates. When the main effect of the factor “group” was significant (p<0.05), subsequent pairwise comparisons of the individual diagnostic groups (healthy controls, MCI-converters, MCI non-converters) were performed. This sequential testing procedure grants, in the case of three groups, that the family-wise alpha-level of 0.05 is retained without correction for multiple testing [40], [42]. Data were analyzed using SPSS 20 (IBM, Armonk, New York). Statistical significance was defined as p<0.05.

A general linear model was applied to MRI data after controlling for possible confounding covariates (age, sex, total intracranial volume) using the statistical package of SPM8.

Voxel-wise GM differences between MCI converters, MCI non-converters, and healthy controls were examined using independent t-test. In order to avoid possible boundary effects, we excluded all voxels with a value of less than 0.1 (absolute threshold masking). Age was entered as a nuisance variable to remove all effects that could be explained by age from the data.

A multiple regression model was applied to detect positive and negative correlations of neuropsychological test results as covariates of interest and age as a nuisance variable on GM differences in all participants. Again, a voxel value of less than 0.1 was excluded, a threshold of p<0.001 and an extended threshold of 100 voxels across the whole brain was used. The significance level was set at a threshold of p<0.001 uncorrected with an extent of 100 voxels across the whole brain to exclude small clusters.

## Results

### Demographic data

At baseline we included 28 cognitively healthy controls (12 men and 16 women) and 27 MCI patients (13 MCI converter, 14 MCI non-converters). The mean (S.D.) age was 71.91 (±6.4) for the MCI subjects and 69.8 (±5.3) for the cognitively healthy controls. The mean follow-up time for clinical and neuropsychological data was mean (S.D.) (±7.7) month. (Further demographic data are presented in table 1– at baseline we did not find any significant group-differences in demographic data and comorbidities.

**Table 1:** Comparison of subject characteristics of MCI converters (converters), MCI non-converters (non-converters) and healthy controls (HC) at baseline.

	HC	Converters	Non-converters	p-value^1^
Number (n)	n = 28	n = 13	n = 14	
	mean±SD	mean±SD	mean±SD	
Age, years	72.2±7.1	73.3±6.7	72.8±7.8	0.552
Gender, n (female %)	16 (57.1)	9 (69.2)	8 (57.2)	0.192
Education, years	9.5±3.7	10.3±4.5	9.6±2.8	0.392
Mean follow-up, month	19.1±8.1	19.3±7.1	19.1±7.7	0.865
ApoE4 carrier, n (%)	8 (28.6)	4 (30.8)	6 (42.9)	0.921
Amnestic MCI, n (%)	---	7 (49.0)	6 (42.9)	0.698^2^
Diabetes mellitus, n (%)	1	2 (9.7)	2 (25.9)	0.425
Hypertension, n (%)	19	10 (71)	11 (70.4)	0.420
Hyperlipidemia, n (%)	4	1 (16.1)	3 (22.2)	0.814

P-values were determined by ^1^Analysis of variance (ANOVA), ^2^student’s independent t-test, SD = standard deviation.

### Results of neuropsychological assessment

Results showed a significant reduction of neuropsychological test results from baseline to follow-up visit in MCI-converters for the following variables using the Wilcoxon test: verbal memory (Z = −4.73, p<0.001), verbal fluency s-words (Z = −3.63, p<0.025), Clox I (Z = −4.75, p<0.002), and Trail A (Z = −2.25, p<0.035). Repeated measures ANOVA revealed group differences for the following neuropsychological test results: MMSE, verbal memory (CERAD), visual memory (CERAD), verbal fluency, naming (BNT), and psychomotor speed (Trail A, Trail B). Pairwise comparison showed a significantly lower score of MMSE, verbal memory (CERAD), visual memory (CERAD), verbal fluency (animals, s-words), naming, and psychomotor speed (Trail A, Trail B) in converters compared to healthy controls. Further, MCI converters compared to MCI non-converters showed worse results of MMSE, verbal memory and naming. Repeated measures ANOVA using time as a factor showed significant group differences for the variables visual memory, verbal fluency s-words, Clox I, and Trail A. Detailed results are presented in Table 2.

**Table 2:** Results of neuropsychological tests at baseline and follow-up.

	Results of neuropsychological tests				Repeated-measures ANOVA^a^					
								Pairwise comparisons		
Neuropsychology	Time point	MCI-c	MCI-nc	HC		Overall		MCI-c vs. MCI-nc	MCI-c vs. HC	MCI-nc vs. HC
						F	p-value			
Number (n)		n = 13	n = 14	n = 28						
		mean±SD	mean±SD	mean±SD						
MMSE	Baseline	25.2±1.7	27.5±1.8	28.6±1.2	group	20.8	p<0.001	p<0.001	p<0.000	p<0.040
	Follow-up	25.0±1.3	27.8±1.9	29.0±1	time	2.49	n.s.	---	---	---
verbal memory^b^	Baseline	15.4±3.2	18.7±3.9	22.3±3.4	group	9.18	p<0.004	p<0.005	p<0.000	p<0.004
	Follow-up	14.5±2.2**	19.7±4.1	22.5±2.6	time	2.97	n.s.	---	---	---
Visual memory^b^	Baseline	5.9±3.9	7.2±2.6	9.6±1.4	group	9.37	p<0.000	n.s.	p<0.000	p<0.004
	Follow-up	4.3±3.4*	8.5±2.6	9.4±1.2	time	8.42	p<0.000	p<0.000	p<0.000	n.s.
Verbal fluency animals	Baseline	21.6±5.2	30.6±8.2	35.8±12.9	group	10.73	p<0.001	n.s.	p<0.001	n.s.
	Follow-up	21.6±5.5	29.9±8.0	36.1±12.9	time	2.45	n.s.	---	---	---
Verbal fluencys-words	Baseline	15.5±8.4	22.2±6.8	24.1±7.3	group	11.82	p<0.005	n.s.	p<0.004	n.s.
	Follow-up	14.7±8.2*	22.5±7.1	24.4±7.3	time	18.92	p<0.002	p<0.020	p<0.001	n.s.
Clox I	Baseline	11.6±2.5	12.7±2.0	12.9±1.8	group	6.37	p<0.017	---	---	---
	Follow-up	10.2±2.8**	13.6±1.1	12.0±3.9	time	8.42	p<0.024	p<0.016	n.s.	n.s.
BNT	Baseline	12.9±1.8	14.2±1.3	14.6±0.9	group	11.72	p<0.001	p<0.030	p<0.001	n.s.
	Follow-up	12.3±1.9	13.9±1.0	14.7±0.5	time	2.12	n.s.	---	---	---
Trail A	Baseline	59.7±16.9	52.8±18.8	36.9±11.4	group	10.37	p<0.005	n.s.	p<0.001	p<0.004
	Follow-up	67.6±32.9*	54.5±16.1	34.4±13.3	time	8.73	p<0.014	n.s.	p<0.004	n.s.
Trail B	Baseline	156.9±105.9	122.6±52.5	91.5±38.6	group	9.27	p<0.013	n.s.	p<0.011	n.s.
	Follow-up	159.9±161.5	138.2±35.3	99.0±53.9	time	3.23	n.s.	---	---	---
GDS	Baseline	10.1±6.1	10.3±6.5	9.8±5.7	group	1.37	n.s.	---	---	---
	Follow-up	9.9±5.2	10.2±5.3	9.9±6.9	time	2.23	n.s.	---	---	---

a Repeated measures ANOVA adjusted for baseline age, education and sex,

b CERAD battery, mean ± standard deviation, healthy controls (HC), MCI-c (MCI converters), MCI –nc (MCI non-converters), Mini-Mental State Examination (MMSE), Boston Naming Test (BNT), Geriatric Depression Scale (GDS), change from baseline to follow-up: **Wilcoxon Test p<0.001, *Wilcoxon Test p<0.05.

### VMB group comparison with T1-weighted images

**Figure 1:**
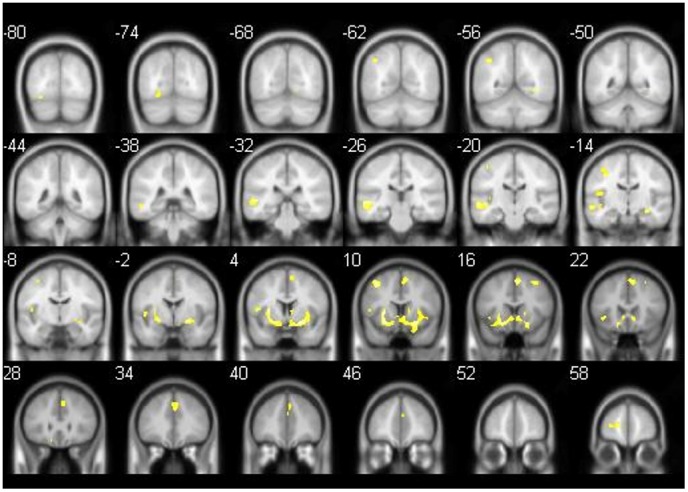
GM loss in MCI converters in comparison to MCI non-converters. T1 Results of comparison of MCI converters and MCI non-converters by using an independent t-test (covariate age, significant clusters: k≥100 voxels/p<0.001 uncorr., threshold 0.1, 6 mm slices). Areas which showed more GM atrophy in MCI converters are superimposed. MCI converters show more GM loss of the frontal lobe bilaterally, the left parietal and temporal lobe, the left putamen, the left insula and the right parahippocampal gyrus.

Following correction for age, MCI-converters in comparison to MCI non-converters were found to have more gray matter atrophy in parts of the left parietal and temporal lobe, the left putamen, the left insula, the right parahippocampal gyrus, and the frontal lobe of both hemispheres (Figure 1, Table 3).

**Table 3:** Shows significant gray matter atrophy in MCI converters in comparison to MCI non-converters.

L/R bAnatomical topography	Cl (*k*)	BA	Peak level^a^		T.c		
			*T*-value	Z	x	y	z
L Inferior Parietal Lobule	147	40	5.38	4.32	−41	−58	43
L Lentiform Nucleus, Putamen	3752	---	5.38	4.32	−27	5	7
L Middle Temporal Gyrus	1187	21	5.34	4.29	−51	−30	1
L Middle Frontal Gyrus	164	6	5.05	4.13	−36	12	48
L Frontal Lobe, Precentral Gyrus	285	9	4.73	3.94	−36	12	40
L Medial Frontal Gyrus	150	10	4.62	3.87	−15	59	6
R Superior Frontal Gyrus	608	8	4.62	3.87	8	18	54
R Medial Frontal Gyrus	527	9	4.41	3.74	5	36	33
R Middle Frontal Gyrus	137	6	4.19	3.60	30	18	52
L Frontal Lobe, Precentral Gyrus	119	44	4.05	3.50	−47	8	9
L Sub-lobar, Insula	195	---	4.15	3.57	−45	−1	1
R Parahippocampal Gyrus	115	19	4.34	3.69	33	−54	−6

a independent *t*-test,

b L = left, R = right, Cluster level (Cl), Brodmann Area (BA), Talairach coordinates (T.c), Significant clusters (k≥100 voxels/p<0.001 uncorr.^a^) showing gray matter loss in MCI converters (n = 13) in comparison to MCI non-converters (n = 14) using analysis of T1-weighted images corrected for age.

Compared to healthy controls, GM loss in MCI converters was mainly located in both temporal lobes, with GM in the left middle temporal lobe, the right fusiform gyrus, and the right inferior and superior temporal gyrus. Within the frontal lobe, only the left frontal lobe was involved. Subsequently, GM loss was found in the lingual gyrus of the right occipital gyrus (For details see Table 4).

**Table 4:** Shows group differences of GM atrophy of MCI converters in comparison to healthy controls.

L/R bAnatomical topography	Cl (*k*)	BA	Peak level^a^		T.c		
			*T*-value	Z	x	y	z
L Middle Temporal Gyrus	1560	21	6.35	5.21	−59	−24	−8
R Temporal Lobe, Fusiform Gyrus	468	19	4.80	4.22	47	−67	−9
R Inferior Temporal Gyrus	386	20	4.06	3.68	63	−25	−24
R Superior Temporal Gyrus	197	38	3.98	3.61	44	26	−23
R Superior Temporal Gyrus	561	38	3.84	3.51	48	12	−29
R Medial Frontal Gyrus	1747	10	4.52	4.02	6	50	10
R Inferior Frontal Gyrus	135	11	4.09	3.70	24	32	−17
R Inferior Frontal Gyrus	199	9	4.04	3.66	48	8	24
R Superior Frontal Gyrus	1063	6	5.09	4.42	8	20	57
R Occipital Lobe, Lingual Gyrus	398	18	3.87	3.53	15	−67	−5

a independent *t*-test,

b L = left, R = right, Cluster level (Cl), Brodmann Area (BA), Talairach coordinates (T.c), Significant clusters (k≥100 voxels/p<0.001 uncorr.^a^) showing gray matter loss in MCI converters (n = 13) in comparison to healthy controls (n = 28) using analysis of T1-weighted images corrected for age.

No significant differences were found between MCI non-converters and healthy controls using a clusters of k≥100 voxels and a significant level of p<0.001 uncorr. Reverse contrasts showed no significant clusters.

### Multiple regression analysis in MCI patients

**Figure 2:**
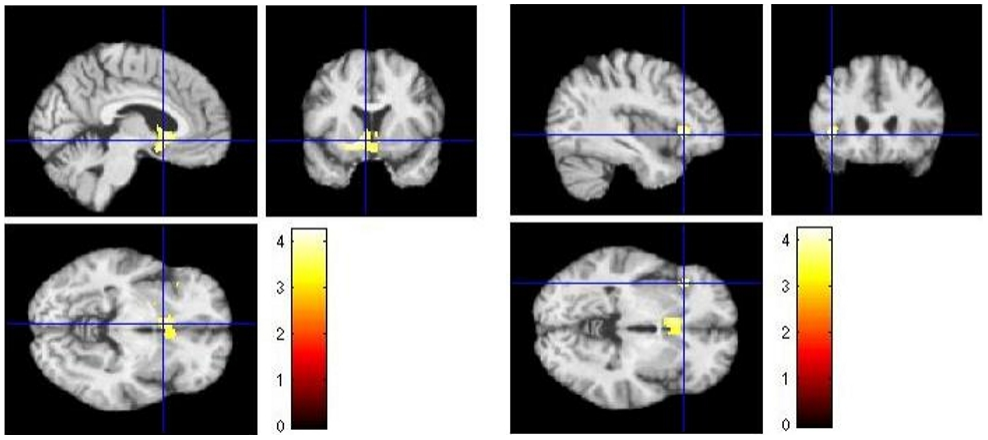
Results of multiple regression analysis with neuropsychological test results. Results of multiple regression analysis of all 55 participants (13 MCI converters, 14 MCI non-converters, 28 healthy controls) adding neuropsychological test results (MMSE, Clox 1, verbal memory, visual memory, Trail A) as covariates. Worse neuropsychological test results for the variables MMSE and verbal memory were positively correlated with higher gray matter atrophy of the left Putamen/Sub-lobar (coordinates *x* = −14, *y* = 8, *z* = −11, *k* = 1163, *T* = 4.26, *Z* = 3.91, *p uncorr*. = 0.000) (left) and the left inferior frontal gyrus (right) (coordinates *x* = −39, *y* = 23, *z* = −2, *k* = 206, *T* = 4.19, *Z* = 3.85, *p uncorr*. = 0.000).

Results of neuropsychological test (Trail A, MMSE, clox 1, verbal memory, and visual memory) as covariate, and age (year) as nuisance variable, were added in a multiple regression analysis using T1- weighted images of all participant, a clusters of k≥100 voxels and a significant level of p<0.001 uncorr. Results showed a positive correlation of neuropsychological test results (MMSE) with gray matter atrophy of the left Putamen/Sub-lobar (coordinates *x* = −14, *y* = 8, *z* = −11, *k* = 1163, *T* = 4.26, *Z* = 3.91, *p uncorr*. = 0.000) and a positive correlation of neuropsychological test results (verbal memory) with gray matter atrophy and the left inferior frontal gyrus (coordinates *x* = −39, *y* = 23, *z* = −2, *k* = 206, *T* = 4.19, *Z* = 3.85, *p uncorr*. = 0.000) – reverse contrast revealed not significant results (For details see Figure 2).

### Analysis of ADC maps reflecting MD

The comparison between the MD of MCI converters and MCI non-converters revealed a significant increase of MD, which were widespread in the gray and white matter of the right frontal lobe including the precentral gyrus (coordinates *x* = 40, *y* = −8, *z* = 30, *k* = 123840, *T* = 6.16, *Z* = 4.65, *p uncorr*. = 0.000). Subsequently, MCI converters showed an increase of MD in the left limbic lobe (coordinates *x* = −6, *y* = −34, *z* = 30, *k* = 202, *T* = 5.11, *Z* = 4.10, *p uncorr*. = 0.000) and the right middle temporal lobe (coordinates *x* = 50, *y* = −12, *z* = −20, *k* = 116, *T* = 5.08, *Z* = 4.09, *p uncorr*. = 0.000). In addition, an increase in MD in MCI converters in comparison to MCI non-converters was found in the basal ganglia and white matter of the parietal, frontal, temporal and occipital lobe.

**Figure 3:**
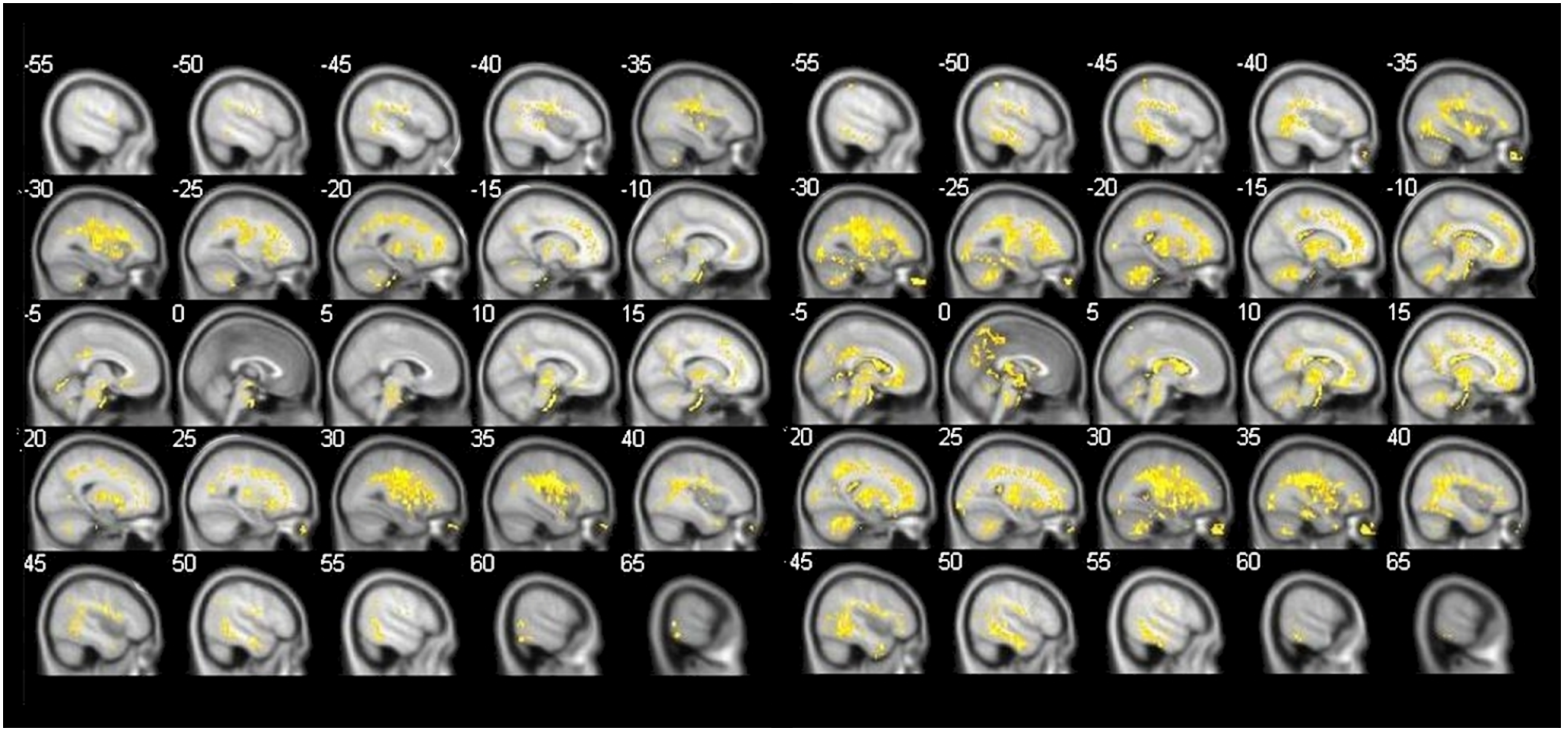
Comparison of ADC values reflecting MD (MCI-converters vs. healthy controls, MCI converters vs. MCI non-converters). left: shows increase in MD in MCI converters in comparison to MCI non-converters. Significant clusters (k≥100 voxels/p<0.001 uncorr.^a^), right: increase in MD in MCI converters in comparison to healthy controls. Significant clusters (k≥100 voxels/p<0.001 uncorr.^a^).

The comparison between MCI converter and healthy controls showed a widespread increase of MD in the white matter of the frontal, parietal, temporal and occipital lobe, and the basal ganglia in MCI converters. An increase of MD in gray matter in MCI converters occurred in the left inferior parietal lobe (coordinates *x* = −52, *y* = −42, *z* = 58, *k* = 109, *T* = 6.07, *Z* = 4.94, *p uncorr*. = 0.000) and in gray and white matter near the right insula (coordinates *x* = 38, *y* = −12, *z* = 24, *k* = 285630, *T* = 5.97, *Z* = 4.88, *p uncorr*. = 0.000) (See Figure 3).

We found no significant group differences in MD when comparing MCI non-converters and healthy controls.

## Discussion

In this study, we examined neuropsychological measures and morphology of GM and WM in patients with MCI and cognitively healthy controls. We found differences in GM atrophy and WM integrity as well as in distinct neuropsychological measures between MCI converters and MCI non-converters. MCI-converters in comparison to MCI non-converters were found to have more GM atrophy in different brain areas. Moreover, MCI converters displayed a widespread increase of MD in WM of the frontal, the parietal, the temporal and the occipital lobe and the basal ganglia compared to stable MCI patients.

### Neuropsychology

Retrospective analysis of the neuropsychological assessment revealed differences in neurocognitive profiles of MCI-converters and MCI-non converters already at baseline. At baseline MCI converters compared to non- converter showed a lower MMSE score and worse results in verbal memory and naming, whereas more cognitive domains were affected at later stages. These results are in line with those of prior studies, which reported lower MMSE scores and deficits in cognitive functions, such as verbal memory or verbal fluency in MCI converters in comparison to MCI non-converters in a comparable retrospective study design [25], [43]. In contrast to prior studies, we found a morphological correlation between neuropsychological deficits in MCI converters and an increased atrophy of GM located in the left frontal gyrus and the left putamen using multiple regression analysis. The left frontal gyrus and its connections to the left putamen are well known to be involved in speech and language functions. The function of areas with GM atrophy corresponds with reported deficits in naming in MCI -converters compared to MCI non-converters at baseline. These findings underline the predictive value of especially asymmetric atrophy pattern in the early stages of dementia.

### VBM analysis

VBM is a well proven neuroimaging analysis technique that allows investigation of focal differences in brain volume, using the statistical approach of statistical parametric mapping. The principle role of VBM is the registration of data sets and the normalization to a standard brain, permitting the evaluation of group-differences. To date, little information is available concerning the use of this method on the brain of a single patient for diagnostic purpose. However, the visualization of patterns in different diseases does not only provide new insights into the pathology and progression, but also guides the diagnostic view of the involved brain regions.

Numerous previous VBM studies have investigated atrophy patterns in patients with AD and MCI and assessed the predictive value of structural brain changes in MCI patients with respect to conversion to AD. Our patient collective comprised 27 MCI patients, whereby the MCI converters were retrospectively identified after a mean follow-up time of about 19.2 months. We detected differences in cerebral GM volume between MCI converters and MCI non-converters at baseline. In our study, the MCI converters exhibited atrophy patterns that are in line with several previous studies [26], [44].

Converters showed reduced GM density of frontal lobe, left parietal lobe, left putamen, left insula, left temporal lobe, and right parahippocampal gyrus in comparison to non-converting MCI patients. Similar to Risacher et al. [25], we found GM atrophy in MCI converters pronounced in the left hemisphere. The GM atrophy found in the left temporal lobe is in accordance to results of a meta-analysis, which reported atrophy of the left temporal lobe as the most consistent structure to predict conversion from MCI to AD [45]. In addition, atrophy of the hippocampus and structures of the limbic system have been reported in MCI patients years before conversion to AD by numerous previous volumetric MRI studies [46], [47]. The significant volume loss in the medial frontal lobe in converters compared to non-converters is partially consistent with previous literature reporting an association of reduced GM in frontal cortex with age-related cognitive decline [48].

### Analysis of ADC maps reflecting MD

Up to now, few studies have analyzed diffusion-weighted images with the methodology of VBM. Differences in GM volume between converters and non-converters are predominately visible in the temporal lobe, whereas MD is increased in several different brain regions that belong to the target regions for volume loss in progressed AD.

Diffusion-weighted imaging is based on thermal molecular movement, which is commonly restricted due to the sophisticated architecture of neuronal tissue. With the help of gradient labeling techniques, the diffusivity of water protons can be quantified. Neuronal loss means disruption of the typical neuronal architecture resulting in an enlargement of the extracellular space and facilitating the diffusion of water protons. Consequently, MD within the affected regions increases and is therefore sensitive to micro structural and pathophysiological alterations in cerebral white and gray matter. We evaluated group differences between MCI converters and MCI non-converters in WM of frontal, parietal, occipital, as well as temporal lobe; the results indicated a loss of white matter integrity in MCI converters. Subsequently, differences in GM integrity were found in the left inferior parietal lobe and the right insula of MCI converters.

Our findings are in line with several previous results showing disrupted WM integrity in the frontal, temporal and parietal lobe as well as subcortical structures in patients with MCI [43], [49], [50]. A prior study employing DWI reported diminished fractional anisotropy in the posterior WM and increased ADC values in the hippocampi in patients with AD compared to healthy controls [51]. Further, results of Smith at al. 2010 using tract-based spatial statistics found a decline of WM tracts in an AD risk study among other regions in the medial temporal lobe and parts of the limbic system [52].

These findings suggest a correlation between an expansion of the extracellular space and neuron loss in the medial temporal lobe in this patient group [53]. The new aspect of our study was the comparison of VBM analyses of both 3D data sets and MD. Our data suggest that WM integrity decline measured by MD can be detected in individuals at high AD-risk, prior to conversion to AD. Therefore, we provide evidence that changes in MD help to detect regions of beginning neurodegeneration and permits a prediction of its extent prior to volume loss. In contrast, we did not find any significant differences, when comparing non-converters and healthy subjects, which further underline the value of MD in pre-clinical AD. However, we found a high number of differences in WM integrity when comparing MCI non-converters and healthy controls. Consequently, the measurement of MD values may have the potential to be established as a biomarker for disease progression from MCI to AD.

### Correlation of neuropsychological variables, VBM data and ADC maps reflecting MD

MCI-converters in comparison to MCI non-converters showed greater GM atrophy in areas such as the middle temporal lobe and medial frontal lobe – both areas are mainly involved in verbal memory and naming. Furthermore, regression analysis, including several neuropsychological test results as covariates, revealed a positive correlation of GM atrophy in the left inferior frontal gyrus and the left putamen with a worse performance in tests assessing frontal-executive functions, psychomotor speed, and memory. These findings are in contrast to other published data which could not find such correlations between neuropsychological test results and GM atrophy of associated brain areas [25], [54]. However, differences in GM atrophy, as well as MD, revealed more significant differences between converting and non-converting MCI patients than neuropsychological results at baseline. Therefore, we suggest a higher diagnostic value of structural MRI data in the early identification of high risk MCI-patients than common neuropsychological tests alone.

### Study limitations

It should be noted that this study had certain limitations. A major limitation is the significantly better neuropsychological results in verbal memory and naming of the MCI non-converters compared to MCI converters at baseline. This could lead to the assumption that the MCI converters were not sufficiently comparable with the MCI non-converters. We could reject this by the fact that both MCI- converters, as well as MCI non-converters, showed significantly worse results for the variable verbal memory in comparison to healthy controls at baseline. Furthermore, all MCI patients fulfilled the diagnostic criteria for MCI according to Petersen et al. [3] at baseline, and the division in MCI converters and MCI non-converters took place retrospectively. On the other hand, our retrospective study design enabled us to study a highly comparable population of MCI converters and MCI non-converters in terms of demographic variables and follow-up period. Another possible limitation of our results is the relatively low mean education of our study population compared to some other studies citied in this paper. To minimize the effect of education on our results, all neuropsychological tests were corrected for age and education and we included the TIV as a covariate in the MRI analysis. Finally, we were faced with the problem that we could not rule out conversion to AD in stable MCI patients post follow-up and our study design compared to prospective designs gives no information on conversion rates from MCI to AD.

## Conclusion

Several key conclusions can be drawn from our results. Firstly, we observed atrophy of GM in MCI converters about 1.5 years prior to conversion to AD. Our results are consistent with previous studies who reported GM atrophy especially in parts of the frontal and temporal lobe in MCI converters [13], [26]. Consequently, our findings confirm that MCI-converters are distinguishable from individuals with MCI who will not convert to AD within the next 1.5 year. In addition, we found significant differences in MD of WM and GM in the left parietal lobe and the right insula in MCI converters compared to MCI non-converters. We suggest that the disturbed integrity of GM and WM reflects an early sign of beginning AD pathology in MCI patients and is jointly responsible for deficits in learning and memory in these patients. This suggestion is supported by our finding that non-converting MCI patients show neither reduced GM volume nor signs of reduced gray and white matter integrity in comparison to healthy controls.
